# Lithotomy, Supra-Pubic Operation

**Published:** 1887-05

**Authors:** Rudolph Menger

**Affiliations:** San Antonio, Texas


### LITHOTOMY---SUPRA PUBIC OPERATION.

*By Dr. Rudolph Menger, San Antonio, Texas.*

For Daniel's Texas Medical Journal.

APRIL 27th, an interesting case of supra pubic lithotomy was performed here by Dr. A. Hahn, assisted by Drs. P. W. Johns, J. Favre, and myself, the result of which I take the liberty to report, in short, as follows:

Patient (male) aged about 36 years, complained, as I understand, for many weeks, and more so two years ago, from symptoms of stone in the bladder, enlarged prostate, cystitis and partial retention of urine. On introducing the catheter and examining per rectum the prostate proved to be hypertrophied and the stone lodging behind the gland, somewhat to the right and upwards of the collum vesicæ. The evacuated urine contained large flocks of mucus and pus—highly albuminous urine. After washing the bladder out thoroughly with antiseptic warm water and introduction of a rubber bag into the rectum, filled with water, and filling the bladder with antiseptic fluid—the incision (about two inches) was made in the linea alba, dividing carefully and under scrupulous cleanliness the deeper tissues, and ligating every small vessel with catgut that came under the knife before reaching the fascia and bladder. On account of the rather small, and, as it seemed, retracted bladder and hypertrophied gland and the situation of the stone, which was firmly impacked in the mucous membrane, it was with much difficulty to reach and feel the end part of the catheter. The bladder was secured with several catgut ligatures and opened without reaching the peritoneum. There was but very little bleeding, considering that the subject was a very unfavorable one for anæsthetising—ether being used at first, and afterwards, chloroform. The stone was a large (mulberry) one, of oval shape, and, as already mentioned, firmly incrustated in the bladder. The operation itself took about two and one-half hours, but, on account of the difficulty in bringing the man under anaesthetics, the entire operation took five hours time. The patient is doing well up to date of writing, two days after operation.

Now the above case is reported mainly on account of its interesting features, and especially because the supra pubic mode of operating is rarely made in America, and perhaps never before in Texas. In Cupple's admirable compilation of statistics of "Texas Surgery," no such operation is recorded, as will be seen from the report of all cases recorded, namely:

Total lithotomies 139, with 121 recoveries and 18 deaths; number of calculi extracted 246; mode of operation, median 45 times; medio-lateral 3 times; lateral 82 times; bi-lateral 2 times and recto-vesical (a 14 oz. stone!) onetime.

